# Nd:YAG Capsulotomy after Phacoemulsification in Vitrectomized Eyes: Effects of Pars Plana Vitrectomy on Posterior Capsule Opacification

**DOI:** 10.1155/2014/840958

**Published:** 2014-04-27

**Authors:** Jong Hwa Jun, Kwang Soo Kim, Sung Dong Chang

**Affiliations:** Department of Ophthalmology, School of Medicine, Dongsan Medical Center, Keimyung University, 56 Dalseong-ro, Jung-gu, Daegu 704-701, Republic of Korea

## Abstract

To compare the progression of posterior capsule opacification (PCO) in patients who required Nd:YAG laser capsulotomy following either combined cataract surgery with pars plana vitrectomy (PPV; C-CV), sequential cataract surgery after PPV (S-CV), or cataract surgery alone (CA). The medical records of 321 patients (408 eyes) who underwent Nd:YAG capsulotomy were retrospectively evaluated. The CA group had a significantly longer time interval from cataract surgery to capsulotomy than that of both the CV group (*P* = 0.006) and the S-CV (*P* = 0.013) and C-CV (*P* = 0.042) subgroups when age-matched comparisons were used. CV patients who implanted a hydrophobic acrylic IOL had shorter time intervals than those of CA patients (*P* = 0.028). CV patients had larger hazard of earlier capsulotomy than CA patients (hazard ratio (HR) = 1.337; 95% confidence interval (CI) 1.100–1.625; *P* = 0.004). C-CV and S-CV patients both had larger hazard than CA patients in earlier capsulotomy (HR = 1.304; 95% CI = 1.007–1.688; *P* = 0.044, HR = 1.361; 95%  CI = 1.084–1.709; *P* = 0.008, resp.). PCO progresses more rapidly in patients undergoing combined or sequential cataract surgery and PPV than in patients undergoing CA.

## 1. Introduction

Cataract formation and progression are common postoperative complications after pars plana vitrectomy (PPV) for a variety of vitreoretinal diseases, including epiretinal membrane and macular hole in phakic patients [1, 2]. Patient age [1, 3], lens opacification before PPV [4], and intravitreal tamponade [5–7] are major risk factors related to cataract progression after PPV. Recently, for cases of vitreoretinal disease combined with moderate to severe lens opacification, combined cataract surgery with PPV has become a routine procedure. The inclusion of cataract extraction leads to better visualization of vitreoretinal structures and earlier visual rehabilitation after PPV can be achieved. However, difficulties with capsulorhexis, higher postoperative inflammation, intraoperative miosis, and corneal edema with Descemet's folds are disadvantages when combining cataract and vitreoretinal surgery [8, 9]. Moreover, in combined surgeries for epiretinal membrane, increased postoperative macular thickness and recurrence of epiretinal membrane have been reported [10].

Posterior capsule opacification (PCO) is the most common complication after cataract surgery [11]. Further, when cataract surgery is combined with PPV, either sequentially or simultaneously, PCO is also the most common factor threatening vision [12]. In previous reports, the rate of PCO after phacovitrectomy has ranged from 10.3% to 51.1% and from 8% to 51% in sequential surgery [12–18]. Particularly, in eyes with vitreoretinal disease, postvitrectromy PCO interferes with the ability to diagnose retinal pathology. Previously, several studies have reported outcomes of phacoemulsification related to PPV; however, the PCO rate was only reported in limited sample sizes and for relatively short follow-up periods. Moreover, it is unknown whether the cataract surgery performed either with or after PPV could influence the interval from cataract surgery to neodymium-doped: yttrium aluminum garnet (Nd:YAG) capsulotomy due to vision threatening PCO. Hence, the aim of this paper was to retrospectively evaluate the records of a large sample of patients over a 14-year period who underwent Nd:YAG capsulotomy for clinically significant PCO with an emphasis on comparing the results of PCO after combined PPV and cataract surgery, sequential PPV and cataract surgery, and cataract surgery alone (CA).

## 2. Materials and Methods

Between January 2000 and December 2012, the medical records of 321 patients (408 eyes) who underwent Nd:YAG laser capsulotomies at the Department of Ophthalmology, Dongsan Medical Center, Keimyung University, Daegu, Korea, were retrospectively evaluated. Patients were divided into two primary groups to investigate whether vitreous removal could influence the progression of PCO; there were 212 eyes from 150 patients in the CA group and 196 eyes from 171 patients in the cataract surgery either with or after PPV group (CV group). 62 patients of CA and 25 patients of CV were enrolled in both eyes. For evaluating capable correlation of outcomes on eyes from same patients, we adopted the point biserial correlation coefficients method. The coefficient of correlation was very low, so we included both eyes of the same patients in this study (data not shown). The CV group was subdivided into two secondary groups: 80 eyes from 71 patients in the combined cataract surgery with PPV group (C-CV group) and 116 eyes from 100 patients in the sequential cataract surgery after PPV group (S-CV group). Patients with a history of uveitis, primary aphakia after cataract extraction, glaucoma filtration surgery, repeated vitrectomy after cataract surgery, intraoperative posterior capsule rupture, combined extracapsular cataract extraction with PPV, and extracapsular fixation of the intraocular (IOL) were excluded. Furthermore, cases who were lost to follow-up for more than 1 year were also excluded to avoid overestimation of the interval to capsulotomy.

Data collection included age at cataract surgery, gender, history of diabetes mellitus (DM), and laterality of surgery. The patient demographics are shown in Table 1. The key parameters of interest were the time interval from cataract surgery to Nd:YAG laser capsulotomy and the IOL materials. For investigating the effects of vitreous on PCO, comparisons were performed between the CV and CA groups. The C-CV and S-CV groups were also compared to test the hypothesis that postoperative inflammation could influence PCO progression. The IOL material subgroups were evaluated for the IOL edge effect and vitreous decompression theory after PPV [17].

### 2.1. Surgical Techniques

All surgeries were performed by one experienced surgeons (KKS) using retrobulbar anesthesia. From 2000 to 2010, conventional 20-gauge, 3-port PPVs were used (Premiere DP 3672 200, Storz, USA, and Millennium Phaco Vitrectomy A/P CX3173, Bausch Lomb, USA) and after October 2011, 23-gauge, 3-port PPVs were used (Millennium Phaco Vitrectomy A/P CX3173). Cataract surgeries were performed with Ten Thousand 10,000 Phacoemulsifier/Aspirator (Alcon Laboratories, USA) from 2000 to 2005 and Millennium Phaco Vitrectomy A/P CX3173 (Bausch Lomb, USA) from 2005 to 2013. Combined cataract and PPV surgeries were performed with continuous curvilinear capsulotomy followed by phacoemulsification and an irrigation/aspiration procedure through the scleral tunnel incision. The PPV was performed without implantation of an IOL. After the PPV, the IOL was implanted in the capsular bag. Routinely, cataract surgeries were performed under retrobulbar anesthesia using a standard clear corneal temporal incision. In a small proportion of cataract surgeries, a scleral tunnel incision method was used. After incision, a continuous curvilinear capsulorhexis was performed, followed by hydrodissection and hydrodelineation. The lens nucleus was removed by phacoemulsification, and the cortical fibers were irrigated and aspirated. The IOL was implanted in the capsular bag and stromal hydration was used for sealing the incision.

### 2.2. Statistical Analyses

The mean age and incidence of diabetes were significantly different between the CA and CV groups (Table 1). As age and diabetes are major risks for progression of PCO, an analysis of covariance (ANCOVA) test was used to allow for age-matched comparisons between groups. In addition, Cox proportional hazards models were used to assess the relative risks of age and diabetes. Fisher's exact test, the independent *t*-test, and one-way analysis of variance (ANOVA) were used to compare the parameters of interest. Mann-Whitney *U* tests and Kruskal-Wallis tests were used to compare IOL subgroups as the data distribution was not normally distributed due to the low sample sizes of the subgroups. The comparison of parameters for the hydrophobic acrylic IOL subgroup for both the surgical method group and the diabetes subgroup within CV was made with independent *t*-tests as the data was normally distributed. Partial correlation analysis was used to assess the relationship between the time interval of the PPV and cataract surgery and the subsequent time interval to capsulotomy. For evaluating Nd:YAG capsulotomy free survival after cataract surgery, a Cox proportional hazards model was employed. A *P* value less than 0.05 was considered to indicate a statistically significant difference.

## 3. Results and Discussion

### 3.1. Results

Of 321 patients, there were 122 men and 199 women. The mean age at cataract surgery was 61.2 ± 0.5 years (range: 31 to 98 years). The mean age of the CA, CV, S-CV, and C-CV groups was 65.1 ± 10.5 years (range: 31 to 98 years), 57.1 ± 9.0 years (range: 38 to 81 years), 57.2 ± 9.6 years (range: 38 to 81 years), and 57.0 ± 8.5 years (range: 40 to 78 years), respectively. The mean age at cataract surgery of the CV group was significantly younger than that of the CA group (*P* < 0.001). There was no difference in the mean age at cataract surgery between the C-CV and S-CV groups (*P* = 0.914). There were also no differences in gender (*P* = 0.093) and laterality proportion (*P* = 0.747). There was a higher proportion of DM patients in the CV group (57.7%) than in the CA group (20.3%) (*P* < 0.001). The S-CV group had a higher proportion of DM patients than the C-CV group (73.8% versus 46.6%) (*P* = 0.000). All comparative statistics are shown in Table 1.

**Figure 1:**
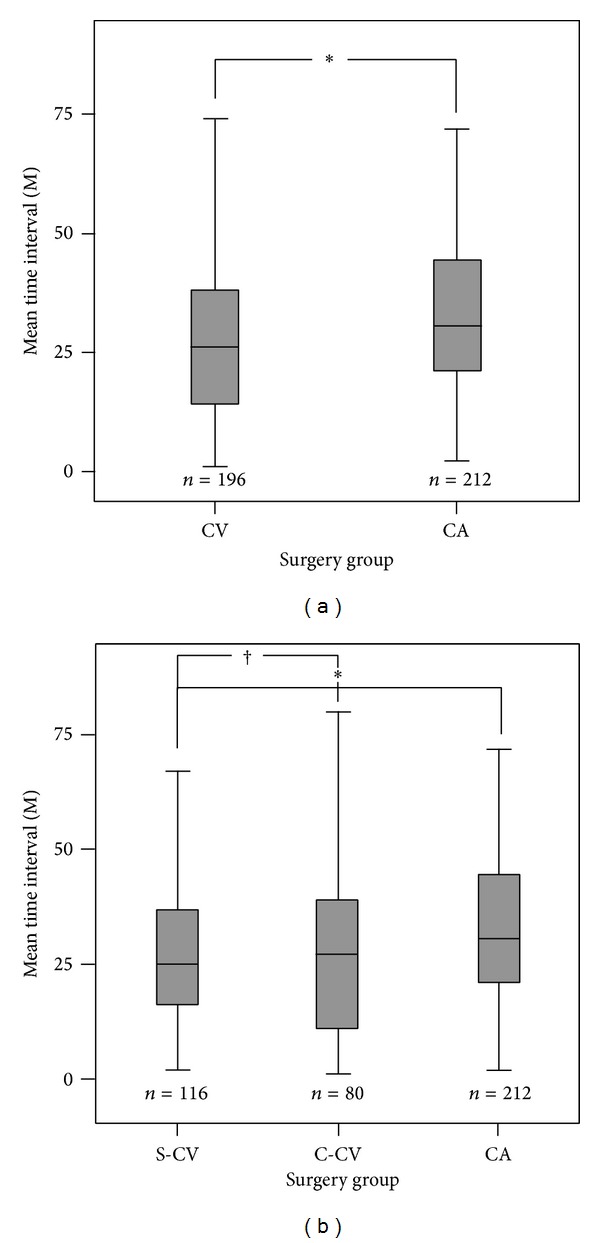
Comparisons of mean time interval from cataract surgery to Nd:YAG capsulotomy in each group (gray bars). The CV group had a shorter time interval than the CA group ((a), *P* = 0.006). The S-CV and C-CV groups had shorter time intervals than the CA group, ((b), *P* = 0.013 and 0.043, resp.). There was no difference between the S-CV and C-CV groups (*P* = 0.321). The asterisk indicates a statistically significant difference in the mean time interval. A cross indicates no difference between S-CV and C-CV groups.

The mean time interval from cataract surgery to Nd:YAG capsulotomy was significantly longer in the CA group than in the CV group (36.3 ± 26.4 versus 29.1 ± 23.1 months; *P* = 0.006). There was also a significant difference in mean time intervals from cataract surgery to Nd:YAG capsulotomy between the three groups when patients were matched for age: 36.3 ± 26.4 months (CA), 29.5 ± 24.4 months (C-CV), and 28.8 ± 22.2 months (S-CV; *P* = 0.023; Table 2). Nd:YAG capsulotomy was performed later in the CA group than in the C-CV group (*P* = 0.013) and the S-CV group (*P* = 0.042; Table 3; Figure 1). In vitrectomized eyes, regardless of the surgical sequence, the clinically significant PCO meant that the mean Nd:YAG capsulotomy free survival time was less than that in nonvitrectomized CA eyes.

**Figure 2:**
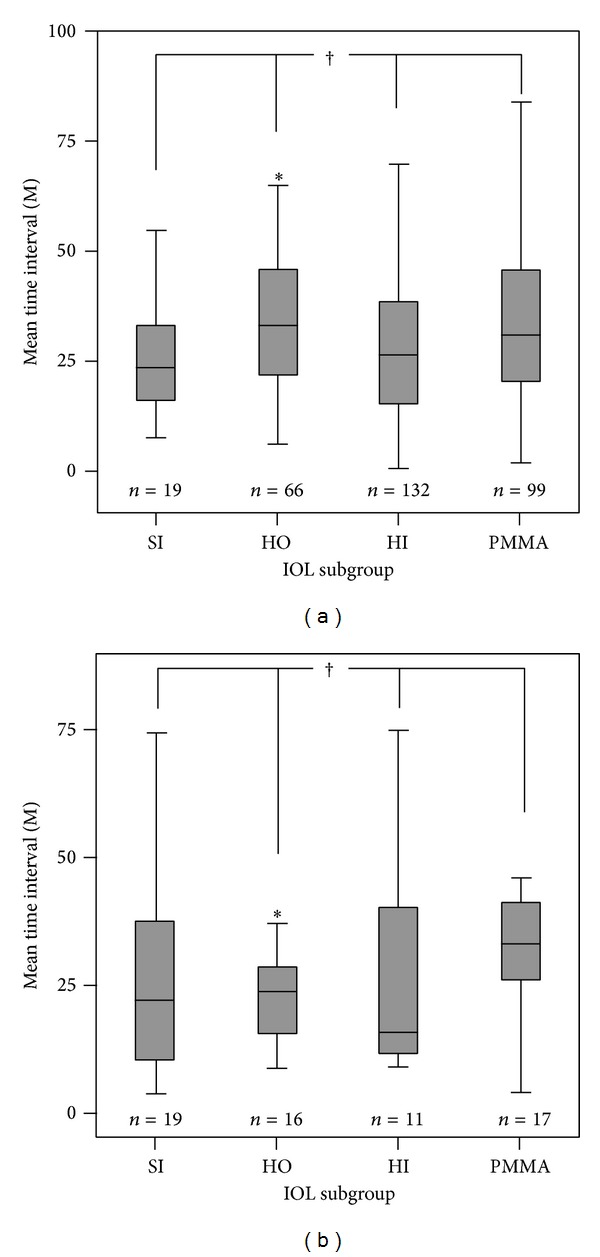
Comparisons of mean time intervals from cataract surgery to Nd:YAG capsulotomy within IOL subgroups. (a) Means comparison of IOL subgroup in the CV group, and (b) means the comparison of the CA group. Within the CV and CA groups, the IOL subgroup showed no difference in the mean time interval to Nd:YAG capsulotomy (*P* = 0.838 and 0.066, resp.). However, the hydrophobic acrylic IOL subgroup within the CV group showed a significantly earlier time to Nd:YAG capsulotomy than that of the CA group (*P* = 0.028). SI, HO, HI, and PMMA refer to silicone, hydrophobic acrylic, hydrophilic acrylic, and polymethyl methacrylate IOL materials, respectively. The asterisk indicates a statistical difference of the mean time interval to capsulotomy between the hydrophobic acrylic IOL subgroups of CV and CA. The cross indicates no difference in the mean time interval of capsulotomy within the IOL subgroup.

Considering the IOL materials of silicone, hydrophobic acrylic, hydrophilic acrylic, and polymethyl methacrylate (PMMA), there were no differences in Nd:YAG capsulotomy free survival times between material types for the CV group (*P* = 0.838). The IOL subgroup for CA eyes showed no difference in Nd:YAG capsulotomy free survival times between material types (*P* = 0.066), but, in the hydrophobic acrylic IOL subgroup, there was a significant difference in the Nd:YAG capsulotomy free survival time between the CA and CV groups. The CA subgroup in which eyes implanted the hydrophobic acrylic IOL showed a longer Nd:YAG capsulotomy free survival time (*P* = 0.028; Table 4; Figure 2).

**Figure 3:**
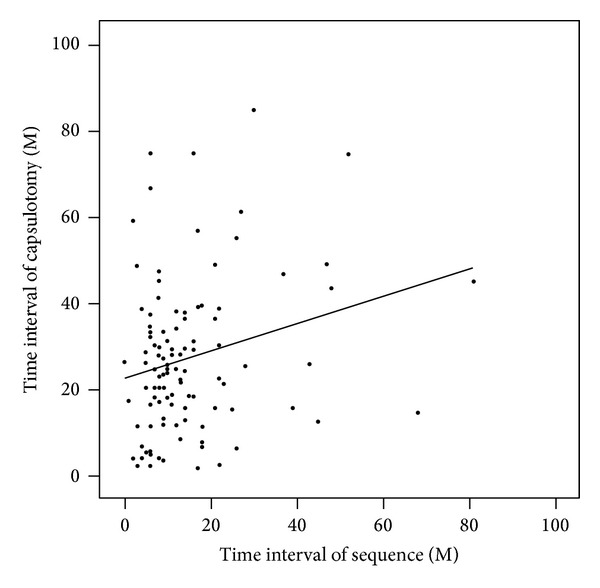
Age-matched partial correlation between the time interval between the PPV and cataract surgeries and the time interval between the cataract surgery and capsulotomy (*P* = 0.009, *r* = 0.248). A longer time interval between PPV and cataract surgeries was associated with a longer time interval from cataract surgery to Nd:YAG capsulotomy.

There were no differences in Nd:YAG capsulotomy free survival times between DM and non-DM patients in either the CA group or the C-CV and S-CV subgroups. Nd:YAG capsulotomy free survival time was not correlated with diabetes history (Table 5). There was a weak yet significant correlation between the time intervals between PPV and cataract surgeries in the S-CV group and the Nd:YAG capsulotomy free survival time (*r* = 0.248, *P* = 0.009; Figure 3).

**Figure 4:**
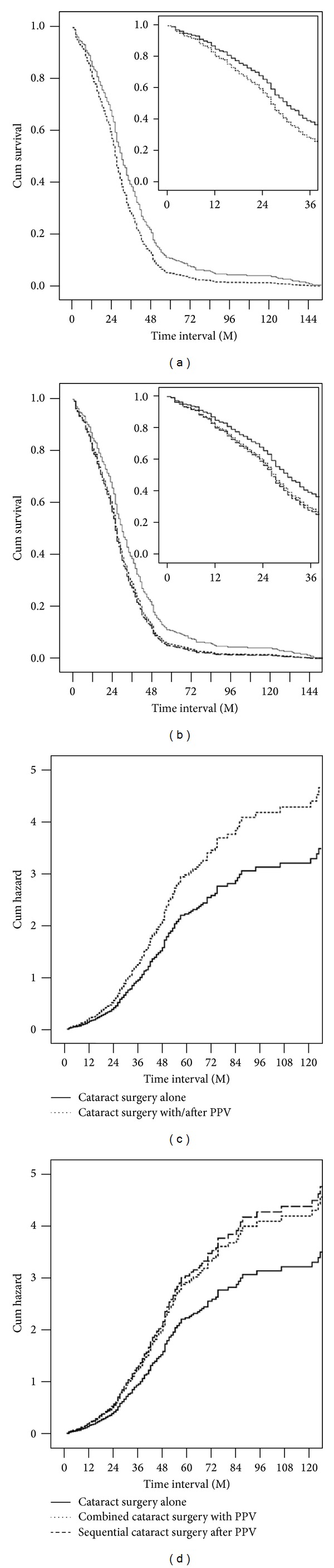
Cox proportional hazards model. Surgical option was identified as a risk factor that influenced the time interval from cataract surgery to capsulotomy. The CV group had a higher risk of a shorter Nd:YAG capsulotomy free survival time than the CA group ((c), hazard ratio (HR) = 1.337; 95% confidence interval (CI) 1.100–1.625; *P* = 0.003). Combined surgery and sequential surgery were associated with an increased risk of a shorter Nd:YAG capsulotomy free survival time ((d), HR = 1.304; 95% CI; 1.007–1.688; *P* = 0.44, HR = 1.361; 95% CI = 1.084–1.709; *P* = 0.008, resp.). The CA group showed a longer Nd:YAG capsulotomy free survival than the CV group (a) and both the C-CV and S-CV groups (b).

The survival curves for each group are shown in Figure 4. The duration of Nd:YAG capsulotomy free survival after cataract surgery showed that the CA group had longer survival than the CV group (*P* = 0.003). Surgical sequence was significantly related to the timing of capsulotomy (*P* = 0.013), but age, DM, laterality of surgery, and gender were not (CV versus CA: *P* = 0.973, 0.875, 0.103, and 0.774; S-CV and C-CV versus CA: *P* = 0.974, 0.855, 0.105, and 0.757, resp.). CV patients had larger hazard of earlier Nd:YAG capsulotomy than CA's by Cox proportional hazard model (hazard ratio (HR) = 1.337, 95% CI 1.100–1.625, *P* = 0.004). Both C-CV and S-CV patients had larger hazard than CA patients in earlier Nd:YAG capsulotomy, respectively (HR = 1.304, 95% CI = 1.007–1.688; *P* = 0.044; HR = 1.361, 95% CI = 1.084–1.709, *P* = 0.008, resp.). There was no difference in hazard between the C-CV and the S-CV groups (*P* = 0.767).

### 3.2. Discussion

Several previous studies have investigated factors associated with PCO. From biochemical investigations, various growth factors, extracellular matrices, integrins, and matrix metalloproteinases are related to lens epithelial cell (LEC) proliferation, migration, and transdifferentiation. Postoperative inflammation in the anterior chamber due to surgical trauma, the IOL design, and surgical technique contribute to PCO formation with progression in cataract surgery [11, 19]. Recently, several studies reported that PCO may occur after cataract surgery performed with or after PPV and causative factors were hypothesized. However, the effects of vitreous body and vitrectomy on PCO progression after cataract surgery are still unknown.

We retrospectively evaluated Nd:YAG capsulotomy free survival times in patients over an extended period, whereas previous studies only focused on the PCO value and rate of laser capsulotomy after sequential or combined surgery. However, the postoperative PCO free duration is also important for patients and ophthalmologists who need to plan post-PPV PCO and vitreoretinal pathology treatments, such as laser photocoagulation or intravitreal injections. The long-term analysis of PCO data should enable an understanding of factors that relate to its progression after cataract surgery with or after PPV.

Toda et al. [17] reported more extensive PCO formation after combined cataract surgery with vitrectomy than CA. In their study, the combined surgery group showed a higher PCO value than that of the CA group in both DM and non-DM patients. They suggested that elevated cytokines caused by postoperative inflammation accelerated the LEC proliferation via autocrine and/or paracrine signaling [17, 20–23]. In addition, Iwase et al. [24] showed a lower PCO rate using 23-gauge phacovitrectomy than when 20-gauge phacovitrectomy was used. They assumed that this was because the 23-gauge phacovitrectomy lowered postoperative inflammation. However, in their study, although there was no difference in the rate of capsulotomy between 23-gauge phacovitrectomy and cataract only surgery, 23-gauge phacovitrectomy showed a higher PCO value than the cataract surgery only group. Rahman et al. assumed that this discrepancy was due to the effect of posterior vitreous pressure on PCO. Furthermore, sequential cataract surgery after vitrectomy has been shown to lead to a higher incidence of PCO than combined surgery [18]. In our study, Nd:YAG capsulotomy free survival times showed no difference in both surgical option groups by both statistical comparison and survival plot. Nevertheless, in sequential surgery, the time interval from PPV to cataract surgery had a weak but highly significant correlation with the time interval from cataract surgery to capsulotomy. It is hypothesized that postoperative inflammation may be related to PCO progression; hence, as the interval from PPV to cataract surgery increases, the post-PPV inflammation decreases.

Theoretically, loss of compression of the vitreous body after PPV may cause early PCO formation in sequential and combined surgeries. Nishi et al. [25] suggested that capsular bend formation is the mainstay of edge effect in square edge IOL, but, according to the loss of compression theory, elimination of the vitreous body causes decompression of the posterior capsule on the optic edge. This phenomenon incurs a loss of acute angle formation in the capsular bag-optic apparatus and more rapid LEC proliferation may occur. In our study, the timing of Nd:YAG capsulotomy after cataract surgery was not dependent on the IOL material in vitrectomized eyes. The only significant difference in Nd:YAG capsulotomy free survival times between vitrectomized and nonvitrectomized eyes was when a hydrophobic acrylic IOL was used. The optic edge technology was applied in hydrophobic acrylic IOL; this indicated that a short Nd:YAG capsulotomy free survival time may be due to early PCO formation mainly caused by a loss of vitreous compression.

It is also possible that there is a change of vitreous circulation after vitrectomy. In the nonvitrectomized state, vitreous circulation of oxygen is limited by the vitreous body, which maintains a relatively low oxygenation state around the lens. Elevated oxygen tension after vitrectomy induces a relatively higher concentration of oxygen distribution near the lens [26, 27]. Therefore, after cataract surgery with or following PPV, relatively higher oxygen tension may be induced near the LECs and more rapid LEC proliferation may occur. As is known from neovascular glaucoma in proliferative diabetic retinopathy, cataract surgery would induce a higher concentration of vitreoretinal factors such as vascular endothelial growth factor near the LEC remnants. This promotes the survival of LECs in a hypoxic state [28] and leads to increased early migration and proliferation in the posterior capsule.

Our study was retrospective in nature, and there were numerous improvements in surgical materials and techniques over the 14-year review period, leading to potential differences in outcomes between the early and late study periods. Furthermore, in present study, there were a thousand or more cases with past history of laser capsulotomy; we decided to include selective cases that could identify at least one-year follow-up observation in medical records. However, with these exclusions, selection bias could occur because the patients who were attended to our Ophthalmology Department would have specific problems like diabetes, concomitant vitreoretinal disease, and other medical disorders. Particularly, patients with vitreoretinal disease would have chronicity of vision problems and would be enthusiastic to regular follow-up observations. Actually, the majority of exclusion was lost to follow-up; their distributions were higher in simple cataract surgery patients. However, our study provides some comprehensive indicators of the pathophysiology of PCO after cataract surgery with or after PPV. It should also be noted that the ages and proportion of patients with DM were different in each group. As age and diabetes may be related to the rate of PCO after phacovitrectomy or cataract surgery only [17, 29], these intergroup differences may have introduced bias into our study. However, the use of age matching reduced the potential influence of this bias and age and diabetes were not identified as risk factors, which has also been noted in a previous study [18]. Furthermore, we retrospectively evaluated the surgical outcomes during longer period, so evolution of surgical techniques and surgical instruments, especially intraocular lenses, could be potential bias of this study. This point would be the limitations of this retrospective study.

## 4. Conclusions

We found that the progressions of PCO are more rapid in patients who underwent combined or sequential cataract surgery and vitrectomy than in patients undergoing CA. For cataract surgery with or after vitrectomy, the formation of postoperative PCO must be considered and appropriate patient counseling and follow-up management should be provided.

## Figures and Tables

**Table 1:** Patient demographics.

Parameter	Group				*P* value
	CA	CV			
		C-CV		S-CV	
Eyes, *n* (patients)	212 (150)	196 (171)			
		80 (71)		116 (100)	

Gender (M/F)	51/99	71/100			0.179*
		35/36		36/64	0.093*

Age (y)	65.1 ± 10.5	57.1 ± 9.0			0.000**
		57.2 ± 9.6		57.0 ± 8.5	***0.000*** ^†^

Laterality (OD/OS)	101/111	101/95			0.488*
		41/39		60/56	0.747*

DM/non-DM	43 : 169	113 : 83			0.000*
		54 : 62		59 : 21	0.000*

Note. CA: cataract surgery alone; CV: cataract surgery with or after pars plana vitrectomy (PPV); C-CV: combined cataract surgery with PPV; S-CV: sequential cataract surgery after PPV; DM: diabetes mellitus.

*Fisher's exact test.

**Independent *t*-test.

^†^One-way ANOVA test.

**Table 2:** Age-matched comparison of the mean time interval from cataract surgery to Nd:YAG capsulotomy.

Parameter	Group			*P* value
	CA	CV	
Mean time interval (M)	36.3 ± 26.4	29.1 ± 23.1		0.006*

	CA	C-CV	S-CV	

Mean time interval (M)	36.3 ± 26.4	29.5 ± 24.4	28.8 ± 22.2	0.023*

*ANCOVA test.

**Table 3:** Time interval differences from cataract surgery to Nd:YAG capsulotomy within subgroups.

	Group		*P* value
Matching of subgroup	CA	C-CV	0.042*
	CA	S-CV	0.013*
	C-CV	S-CV	0.321*

*Contrast test after ANCOVA test.

**Table 4:** Comparison of the mean time interval within the IOL subgroup in cataract surgery with or after vitrectomy and cataract surgery alone.

	Subgroup				*P* value
	Silicone	Hydrophobic acrylic	Hydrophilic acrylic	PMMA	
Mean time interval (M)					
CV	30.7 ± 29.3	29.1 ± 21.6	25.8 ± 19.9	27.1 ± 20.7	0.838*
CA	36.4 ± 25.3	35.5 ± 22.1	22.6 ± 8.7	32.8 ± 15.9	0.066*
*P* value	0.084**	***0.028*** ^†^	0.909**	0.378**	

Note. IOL: intraocular lens; PMMA: polymethyl methacrylate.

*Kruskal-Wallis test.

**Mann-Whitney *U* test.

^†^Independent *t*-test.

**Table 5:** Comparisons of the mean time interval from cataract surgery to Nd:YAG capsulotomy within the PPV subgroup.

Parameter	Subgroup		
	CA	C-CV	S-CV
Mean time interval (M)			
DM	29.7 ± 22.7	32.0 ± 22.8	27.6 ± 22.6
non-DM	28.2 ± 23.8	26.0 ± 21.6	34.7 ± 29.0
*P* value	0.654*	0.152*	0.323*

*Independent *t*-test.
